# pH-Responsive Water-Soluble Chitosan Amphiphilic Core–Shell Nanoparticles: Radiation-Assisted Green Synthesis and Drug-Controlled Release Studies

**DOI:** 10.3390/pharmaceutics15030847

**Published:** 2023-03-05

**Authors:** Thananchai Piroonpan, Pakjira Rimdusit, Saowaluk Taechutrakul, Wanvimol Pasanphan

**Affiliations:** 1 Center of Radiation Processing for Polymer Modification and Nanotechnology (CRPN), Faculty of Science, Kasetsart University, Bangkok 10900, Thailand; 2 Department of Materials Science, Faculty of Science, Kasetsart University, Bangkok 10900, Thailand

**Keywords:** water-soluble chitosan, water radiolysis, amphiphilic, core–shell, controlled release, paclitaxel berberine

## Abstract

This work aims to apply water radiolysis-mediated green synthesis of amphiphilic core–shell water-soluble chitosan nanoparticles (WCS NPs) via free radical graft copolymerization in an aqueous solution using irradiation. Robust grafting poly(ethylene glycol) monomethacrylate (PEGMA) comb-like brushes were established onto WCS NPs modified with hydrophobic deoxycholic acid (DC) using two aqueous solution systems, i.e., pure water and water/ethanol. The degree of grafting (DG) of the robust grafted poly(PEGMA) segments was varied from 0 to ~250% by varying radiation-absorbed doses from 0 to 30 kGy. Using reactive WCS NPs as a water-soluble polymeric template, a high amount of DC conjugation and a high degree of poly(PEGMA) grafted segments brought about high moieties of hydrophobic DC and a high DG of the poly(PEGMA) hydrophilic functions; meanwhile, the water solubility and NP dispersion were also markedly improved. The DC-WCS-PG building block was excellently self-assembled into the core–shell nanoarchitecture. The DC-WCS-PG NPs efficiently encapsulated water-insoluble anticancer and antifungal drugs, i.e., paclitaxel (PTX) and berberine (BBR) (~360 mg/g). The DC-WCS-PG NPs met the role of controlled release with a pH-responsive function due to WCS compartments, and they showed a steady state for maintaining drugs for up to >10 days. The DC-WCS-PG NPs prolonged the inhibition capacity of BBR against the growth of *S. ampelinum* for 30 days. In vitro cytotoxicity results of the PTX-loaded DC-WCS-PG NPs with human breast cancer cells and human skin fibroblast cells proved the role of the DC-WCS-PG NPs as a promising nanoplatform for controlling drug release and reducing the side effects of the drugs on normal cells.

## 1. Introduction

Green and versatile processes have been designed towards green nanotechnology, which is a promising platform for application in various biomedical and healthcare applications, particularly as nanocarriers for controlled release systems. Several aspects, such as one-pot synthesis, water-based synthesis, water-soluble materials, and simultaneous sterilization under last-step synthesis, can be considered for healthcare product preparation. For controlled release characteristics, it is particularly important to govern drug encapsulation and to control release behavior over a prolonged period in biologically water-based systems and operating sites. The drugs, such as anticancer drugs or antibiotics, generally have low bioavailability (due to low water solubility) and low stability. This issue seriously limits further clinical use of the drugs to achieve high treatment efficiency. Enhancing the solubility of water-insoluble drugs, such as paclitaxel (PTX) and berberine (BBR), has received significant attention [1,2]. Efforts have been made towards the design of sophisticated drug structures and formulations using various nanomaterials, such as liposome and lipid micelles [3], nanoparticles (NPs), nanocapsules [4], and amphiphilic core–shell nanoparticles [5]. Polymeric core–shell nanoparticles can be fabricated from a single polymer or multiple polymeric building blocks of copolymers or graft copolymers comprising two or more components, which self-assemble into core–shell NPs [6]. The core–shell NPs can be applied as a drug nanocarrier in drug delivery processes. Among various types of nanocarriers, amphiphilic NPs are a popular platform for carrying water-insoluble drugs. They consist of a hydrophobic inner core and a hydrophilic outer shell with a size of about 5–150 nm [7,8,9]. The hydrophobic core compartment encapsulates water-insoluble drugs; meanwhile, the hydrophilic shells protect aggregation and increase water dispersion of the nanocarriers. For this reason, scientists are interested in the development of amphiphilic NPs for improving the efficiencies of drug loading, controlled drug release, and clinical treatment. As part of the evolution of amphiphilic core–shell NPs being used for drug delivery systems, biocompatible, biodegradable, and nontoxic polymers have been considered. Particularly, natural polymers such as poly(L-lysine) [10], poly(γ-glutamic acid) [11], pullulan [12], dextran [13], and chitosan [14] have received much attention for potential use in the construction of nanocarriers in this field [15].

Chitosan (CS) is a hydrophilic and cationic natural polymer exhibiting unique properties due to its chemical structure and functionalities. CS exhibits biodegradability [16], biocompatibility [17], low toxicity [18], and functionalization ability. With the amino (–NH_2_) and hydroxyl (–OH) groups, their functions enable CS to conjugate with several molecules, such as ethylene glycol (PEG) [19,20,21], gallic acid [22], deoxycholic acid [23], and bombesin peptide [24]. According to the functional groups, CS also has metal chelation and reduction reaction capacities [25,26], antioxidant/antimicrobial activities [27], and pH-responsive ability [28]. With its unique characteristic, CS has been applied as a main natural polymer for the development of several biomaterials, such as pH-responsive hydrogels for delivering 5-fluoro uracil [29,30] and nanocarriers for delivering genes, proteins, DNA, siRNA, and anticancer drugs [31]. Although CS is a hydrophilic polymer in nature, it is insoluble in water and neutral pH media because its pKa value is ~6.5. Accordingly, solubility is a limitation of CS in various applications, including biomedicine. Therefore, the modification of CS through conjugation with different hydrophobic and hydrophilic molecules towards the appropriate amphiphilic derivatives has been continuously developed. Numerous hydrophobic molecules, such as steric acid [32], cholic acid [33], and deoxycholic acid [23], have been used for preparing hydrophobic CS derivatives. As mentioned, CS is hydrophilic polymer; however, hydrophobic modified CS brings about noticeable agglomeration [23]. Thus, improving water solubility and the dispersion of hydrophobic modified CS is an important route to accomplishing realistic amphiphilic CS with good water dispersion. Chemical modification of CS with various hydrophilic and hydrophobic molecules to improve the solubility of CSNPs in the delivery system has been reviewed in previous reports [14,34]. PEG is the most widely used hydrophilic molecule for chemically modifying CS to improve solubility and biocompatibility. PEG is appropriate for biological applications due to its nontoxic, biodegradable, biocompatible, immunogenic, and nonirritant hydrophilic properties that prolong circulation in blood systems [19,20,21]. Deoxycholic acid (DC) is one of the hydrophobic molecules that has been approved by the FDA (in 2015) for the treatment of submental fat to improve aesthetic appearance and reduce facial fullness or convexity. Thus, DC has been selected as the hydrophobic core of nanocarriers when tailoring CS for drug delivery applications. DC modified onto irradiated CS colloids has been proposed to control and lower the particle size of CSNPs to as small as 30–50 nm [23]. Nevertheless, hydrophobic DC-modified CSNPs of small particle size were obtained, and unstable and agglomerated NPs within a short period were underlined. The high molecular weight of the CS backbone (ca. >300–500 kDa) and the hydrogel bond interaction of the outer shells of the CSNPs could be the reason for poor solubility and agglomeration. Thus, modifying CS with only hydrophobic DC does not achieve practical and stable amphiphilic NPs in water-based biological media. 

Besides chemical functionalization methods, lowering the MW of CS towards water-soluble species has also been developed through the use of a radiation-induced chain scission process [35]. We expect low MW water-soluble CS (WCS) used as natural polymer backbone to improve the grafting efficiency, water-solubility/dispersion, and drug encapsulation efficiency of the amphiphilic core–shell CSNPs, thus achieving desirable nanocarrier characteristics for drug delivery systems. In addition, radiation-induced graft copolymerization is also an excellent method for tailoring CS towards becoming a multifunctional grafted copolymer building block for NP self-assembly. Due to being catalyst- and initiator-free, as well as a simple, effective, and green process, radiation-induced graft copolymerization is an acceptable method for the synthesis and modification of advanced biomaterials [36,37]. In a very clean water-based system, the irradiation technique can provide a robust grafted segment that provides a high amount of hydrophilic function, thus leading to enhanced water dispersion of the final amphiphilic NP product. Up to now, although several efforts have been made to design amphiphilic NPs, challenges related to tailoring WCS with comb-shaped poly(PEGMA) hydrophilic shells through an irradiation process have not yet been addressed or clarified regarding controlled drug release applications. With the renovated combination of WCS and robust grafted poly(PEGMA) brushes, the obtained amphiphilic core–shell WCS NPs would offer desirable properties for controlled drug release systems in different aspects, such as water solubility and dispersion, encapsulation efficiency, controlled release behavior, pH-responsive function, and synergistic bioactivities. 

In this research, we aim to propose an original work by developing DC-conjugated water-soluble chitosan and robust grafted poly(PEGMA) comb-like brushes as a multicompartment grafted copolymer building block for the self-assembly of amphiphilic core–shell NPs. Herein, WCS was used as the main natural polymer for enhancing DC conjugation ability and improving the dispersion of the final amphiphilic NP products. The influences of the main CS and WCS polymer types, the absorbed doses, PEGMA monomer concentrations, and the solvent system on the degree of grafting (DG) were studied. Confirmation of successful synthesis and the characteristics of the polyPEGMA-DC-WCS (DC-WCS-PG) building block and its self-assembly were assessed by DG, FT-IR, ^1^H NMR, XRD, AFM, TEM, and water solubility/dispersion properties. The water-insoluble drug (i.e., PTX and BBR) encapsulation and controlled release behaviors of the DC-WCS-PG NPs under a pH of 6.5 and 7.4 were investigated. In vitro cytotoxicity of drug-loaded NPs was compared with free drugs, and the neat NPs were also assessed with breast cancer cells and non-cancerous cells. 

## 2. Materials and Methods

### 2.1. Materials

Chitosan (CS) flake (degree of deacetylation: %DD = 90 and M_w_~300 kDa) was acquired from Seafresh Chitosan (Lab) Co., Ltd. (Bangkok, Thailand). Deoxycholic acid (DC) was purchased from Nacalai Tesque (Kyoto, Japan). Poly(ethylene glycol) monomethacrylate (PEGMA, MW = 486 g/mol) was obtained from Polysciences, Inc. (Warrington, PA, USA). 1-Ethyl-3-(3′-dimethylaminopropyl) carbodiimide (EDC) was purchased from TCI (Kawaguchi, Japan). *N*-hydroxysuccinimide (NHS) was purchased from Tokyo Kasei Kogyo Co., Ltd. (Kawaguchi, Japan). Paclitaxel (PTX, MW = 853.90 g/mol) was purchased from Calbiochem (Darmstadt, Germany). Berberine chloride hydrate (BBR, MW = 336.36 g/mol) was obtained from Sigma-Aldrich (St Louis, MO, USA). Acetic acid (CH_3_COOH) and ethanol (CH_2_OH) (AR grade) were purchased from Labscan, Co., Ltd. (Bangkok, Thailand). Dialysis membranes (MWCO = 1000 Da, 6000–8000 Da, and 12,000–14,000 Da) were purchased from Membrane Filtration Products, Inc. (Seguin, TX, USA). All chemicals were used without further purification.

### 2.2. Instruments and Characterizations

Gamma-ray irradiation was carried out using a ^60^Co source in a Gammacell 220 irradiator with a dose rate of 4.98 kGy/h at room temperature. Fourier transform infrared (FTIR) spectra were recorded in the absorption mode using a Bruker Tensor 27 FTIR spectrometer (Philadelphia, PA, USA). The FTIR spectra were detected in the frequency range of 4000–650 cm^−1^ through 32 scans at a resolution of 2 cm^−1^. Elemental analysis was conducted on a LECO CHNS-932 instrument with a combustion temperature of 950 °C under air atmosphere with O_2_ as a combustion gas (flow rate 20 mL/min) and He gas as a carrier (flow rate 200 mL/min). Proton nuclear magnetic resonance (^1^H NMR) spectra of samples in D_2_O solution were obtained on a Bruker Avance III NMR spectrometer (500 MHz). X-ray diffraction (XRD) patterns were collected by a Bruker AXS (Karlsruhe, Germany) D8 Advance X-ray diffractometer. The sample was prepared on a polymethyl methacrylate (PMMA) specimen holder ring. XRD measurements were performed with Cu Kα radiation as an X-ray source operating at 50 kV and 100 mA. The XRD scanning counts were accumulated every 0.0389° (2θ) over 8° to 28° (2θ). Transmission electron microscope (TEM) images were obtained on a Hitachi (Tokyo, Japan) H-7650 and HT 7700 at 100 kV. The sample was diluted to a certain concentration (1 µg/mL). Vigorous stirring and sonication were performed before dropping the solution onto a carbon-coated copper grid. The sample was dried in a desiccator for 24 h before TEM measurement. Atomic force microscope (AFM) measurements were performed with a NanoWorld (Neuchâtel, Switzerland) NCHR-50 using a Point probe Silicon-SPM-Sensor with a resonance frequency of 320 kHz and a force constant of 42 N/m. Sample solution (1 µg/mL) was dropped onto a mica slide and dried in a desiccator for 24 h before AFM measurement. 

### 2.3. Preparation of Water-Soluble Chitosan (WCS) Nanoparticles

The WCS was prepared following the procedure outlined in our previous work [35]. Briefly, CS (10 g) was dissolved in aqueous acetic acid (1% *v/v*, 1000 mL) under continuous magnetic stirring. The CS solution was gamma-irradiated with a dose of 80 kGy at ambient temperature. Subsequently, the sample was neutralized in NaOH solution (1% *w/v*, 600 mL) via the precipitation method. Sodium acetate was separated from the solution using a dialysis membrane (MWCO = 1000 Da). The dialyzed solution was frozen and lyophilized using a freeze dryer system at −40 °C to a constant weight to achieve water-soluble chitosan (WCS). 

### 2.4. Preparation of Deoxycholate-Functionalized Water-Soluble Chitosan (DC-WCS) Nanoparticles

WCS (0.2 g) was dissolved in distilled water (70 mL). DC (0.4814 g, 1 mol equiv. to WCS) was gently dissolved in an ethanol solution. EDC (0.2351 g, 1 mol equiv. to WCS) and NHS (0.1411 g, 1 mol equiv. to WCS) were gradually added into the DC solution in an ice bath and stirred for 15 min. The WCS solution was poured into the mixture of DC/EDC/NHS. The reaction mixture was carried out at room temperature for 24 h. The sample was dialyzed (MWCO = 1000 Da) in distilled water, with water renewed over a period of 48 h. The dialyzed solution was lyophilized to a constant weight by a freeze dryer at −40 °C for 24 h to obtain deoxycholate-functionalized water-soluble chitosan (DC-WCS). The DC-functionalized non-water-soluble chitosan (DC-CS) was prepared according to our previous work [23,38] Briefly, CS (0.1631 g, 1 mol) was pre-dissolved in NHS (0.2303 g, 1 mol equiv. to CS) with distilled water (30 mL) and stirred for 0.5 h. DC (0.7851 g, 1 mol equivalent to CS) and EDC (0.3834 g, 1 mol equivalent to CS) in a methanol solution were then added to NHS-dissolved chitosan to obtain DC-CS. Purification was carried out using the same procedure. 

### 2.5. Preparation of PEGMA-Grafted DC-WCS

DC-WCS powder dispersed in an ethanol solution (0.05% *w/v*, 125 mL) was mixed with an aqueous solution of PEGMA (0.30, 0.89 and 1.50% *w/v*, 125 mL) to achieve a series of DC-WCS:PEGMA with mole ratios of 1:1, 1:3, and 1:5 (or PEGMA concentrations of 0.15, 0.445, and 0.75% *w/v*, respectively) in single-distilled water. The mixtures of DC-WCS and different PEGMA concentrations (0.15, 0.445, and 0.75% *w/v*) were gamma-irradiated (doses of 2, 10, 20, and 30 kGy). The irradiated samples were transferred into dialysis bags (MWCO = 12,000–14,000 Da) enclosed in 2 L of distilled water and were dialyzed with renewed water for 48 h. The samples were lyophilized in a freeze dryer at −40 °C to obtain DC-WCS-PG. The degree of grafting (DG) of PEGMA on the DC-WSC was calculated according to the following relationship: [(*W* − *W*_o_)/*W*_o_] × 100, where *W*_o_ and *W* are the initial weight of DC-WSC (non-grafted) and the weight of the grafted DC-WCS-PG, respectively. Similarly, the ethanol/water (1:1 *v/v*) co-solvent was used as the reaction solvent instead of the single-distilled water, and the reaction was carried out under the same procedure. In addition, PEGMA-grafted DC-CS (DC-CS-PG) was prepared as in a previous work [38], and it was used for comparative study with the DC-WCS-PG. 

### 2.6. Water Solubility Test

The water solubilities or water dispersion abilities of the DC-WCS-PG NPs were observed by measuring light transmission at a wavelength of 600 nm [22,39]. The samples, with amounts of 0.2, 10, 15, 20, 25, 30, 35, 40, 45, and 50 mg, were dissolved in distilled water (10 mL) to achieve final concentrations of 0.02, 1.0, 1.5, 2.0, 2.5, 3.0, 3.5, 4.0, 4.5, and 5.0 mg/mL. The percent transmittance of each solution was measured by a UV-spectrophotometer at a visible wavelength of 600 nm. Similarly, the percent transmittance of WCS and DC-WCS in water and DC-WCS in ethanol were also assessed using the same procedure. 

### 2.7. Drug Encapsulation Efficiency Studies

The aqueous solution of the sample of DC-WCS-PG NPs (2 mg/mL, 1 mL), prepared using a WCS:PEGMA ratio of 1:5 and at a dose of 10 kGy (DG = 252%), was mixed with BBR in an ethanolic solution (2 mg/mL, 50, 150, 250, 350, and 450 µL). The mixture volumes were adjusted by adding ethanol to obtain final BBR concentrations of 0.1, 0.3, 0.5, 0.7, and 1.0 mg/mL, respectively. Each mixture was softly stirred at room temperature for 24 h. The BBR-loaded DC-WCS-PG NPs were separated from the solution by centrifugation at 10,000 rpm for 30 min. The supernatant was collected for UV-vis absorption measurement at a wavelength of 350 nm. Drug encapsulation efficiency (EE, %) was determined from [(A − A_o_)/A_o_] × 100, where A and A_o_ are the absorbance at 350 nm [40] of the remaining amount of BBR in the supernatant after encapsulation and the initial amount of BBR before encapsulation, respectively. Drug loading content (LC, %) was also calculated from [(*W*_D_ − *W*_o_)/*W*_o_] × 100, where *W*_D_ and *W*_o_ are the weight of BBR-encapsulated NPs and the initial weight of NPs, respectively. Similarly, PTX was also encapsulated into DC-WCS-PG NPs using the same protocol, and the UV-vis absorption of PTX was observed at 230 nm [41,42]. All samples were performed in triplicate and data were reported as mean values ± SD. Similarly, the encapsulation of free BBR and PTX was also performed in the PG-DC-CS NPs using the same procedure.

### 2.8. In Vitro Controlled Release Studies

The drug-controlled release studies were carried out using the dialysis method according to previous reports [43,44,45] with some modification. The dynamic drug release studies were performed in phosphate buffered saline (PBS) at a pH of 6.5 and 7.4 and incubated at 37 °C. The PTX- and BBR-loaded DC-WCS-PG NPs (500 mg) were suspended in PBS (3 mL) and transferred into a dialysis membrane (MWCO ~6000–8000 Da). Samples in the dialysis membrane were immersed in PBS buffer (25 mL) and stirred in a shaking water bath at 37 °C. Samples in the outer membrane (1 mL) were periodically removed, filtered, and assayed. The same volume of fresh medium was replaced into each sample after solution withdrawal. Study of free BBR release behavior was carried out with the same procedure by replacing BBR-loaded DC-WCS-PG NPs inside the dialysis membrane with a free BBR solution. The amount of released BBR was analyzed with a UV-vis spectrophotometer at 350 nm. BBR release studies were performed in triplicate for each sample. Drug release (%) was determined from [*A*_t_/*A*_i_] × 100, where *A*_t_ is the absorbance of drug release at time t and *A*_i_ is the absorbance of the initial drug. Similarly, a PTX-controlled release study was also carried out using the same protocol, with the UV-vis absorption of PTX measured at the wavelength of 230 nm.

### 2.9. Antifungal Behavior Observation 

*S. ampelinum,* from infected untreated grape leaves at Amphoe Pak Kret, Nonthaburi province, Thailand, was isolated according to a previous report [46]. The *S. ampelinum* was obtained from the Department of Plant Pathology, Faculty of Agriculture, Kasetsart University (Thailand). The *S. ampelinum* (1–2 days old) was applied onto a potato dextrose agar plate and treated with 10, 100, 1000, 10,000, and 100,000 ppm of pure BBR and BBR-loaded DC-WCS-PG NPs (as prepared in the previous section). The diameters of the inhibition zone were measured. All samples were performed in triplicate and the data were reported as mean values ± SD. Percent inhibition zone expansion was calculated from [(*I*_t_ − *I*_0_)/*I*_0_] × 100, where *I*_t_ and *I*_0_ are the diameters of the inhibition zone (clear zone) after treatment at time t and at the initial time (at 24 h).

### 2.10. Cytotoxicity Test

Cytotoxicity tests were conducted with breast cancer and human skin fibroblast cells. The human breast adenocarcinoma cell line MCF-7 or breast cancer cells were applied for cytotoxicity study using the MTT assay. The MCF-7 breast cancer cells were maintained in Dulbecco’s Modified Eagle’s Medium (DMEM) with 10% fetal calf serum (FCS) and incubated at 37 °C under an atmosphere of 8% CO_2_ in tissue culture flasks [47]. After cells had grown to a desired concentration (~80% confluence), a concentration of 40,000 cells/mL of MCF-7 breast cancer cells was seeded in each well of a 96-well plate. The cells were incubated overnight to allow cells to adhere onto the surface of the plate, and the media was then removed. The cell samples were then re-suspended in DMEM and treated with PTX-loaded DC-WCS-PG NPs (DEE ~80%). The samples were incubated for 24 h and cellular morphologies were investigated using bright-field microscopy. After cellular investigation, MTT dye in PBS solution (5 mg/mL, 10 µL) was added into the cell samples and incubated at 37 °C for 3 h. The formazan crystals in the MTT solution were removed by adding 100 µL of dimethyl sulfoxide (DMSO) into the plates, and the sample was further incubated for 20 min. The absorbance of the plates was read at a wavelength of 570 nm using a SpectraMax M2 microplate reader. Similarly, the cytotoxicity test of pure PTX was also carried out using the same procedure. In addition, human skin fibroblast cell (CRL 2522) preparation and cytotoxicity tests were performed using the same procedures. 

## 3. Results and Discussion

**Scheme 1:**
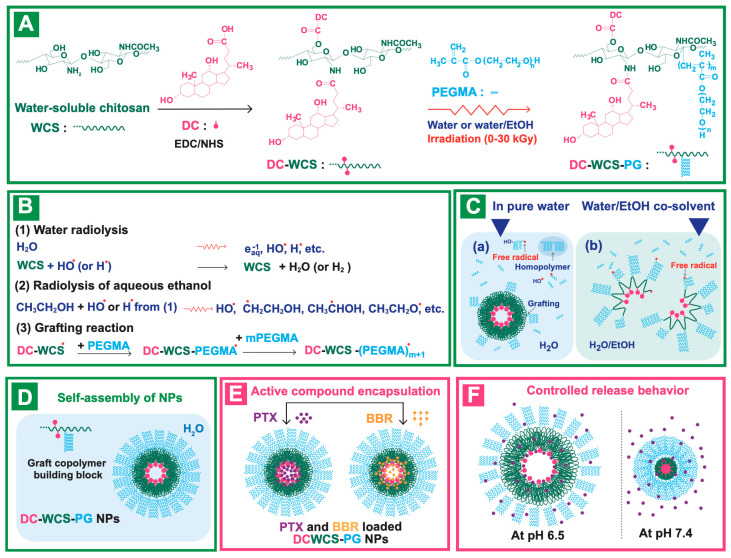
(**A**) Synthesis route of the DC-WCS-PG graft copolymer. (**B**) Radiolysis mechanism and radiation-induced grafting of PEGMA onto DC-WCS in aqueous solution. (**C**) Possible behavior of the radiation-induced grafting of PEGMA onto DC-WCS in (**a**) water and (**b**) an aqueous water/ethanol co-solvent. (**D**) DC-WCS-PG graft copolymer building block and its self-assembly into amphiphilic core–shell NPs in water. (**E**) PTX and BBR encapsulation in DC-WCS-PG NPs. (**F**) Possible drug release behavior at pH 6.5 and 7.4.

The synthesis of grafted copolymer building blocks for the construction of amphiphilic core–shell WCS NPs was performed in a water-based system, as shown in Scheme 1A. The low MW of WCS (M_w_ ~5000 Da, PDI ~1.26) was prepared in an aqueous solution using radiation-induced chain scission as described in previous studies [26,35]. To provide the hydrophobic core structure, DC molecules were chemically conjugated onto WCS. Scheme 1A shows the conjugation of DC onto WCS in the presence of EDC carbodiimide and an NHS coupling agent. Basically, EDC and NHS initiate the reactive ester species on DC, and the nucleophilic reaction can appear at –NH_2_ and/or –OH, thus leading to amide and/or ester bond formation, respectively. The conjugated DC-WCS products were obtained.

For decorating the DC-WCS with the hydrophilic poly(PEGMA) brushes, two types of aqueous solution systems (pure water and water/ethanol co-solvent) were applied for radiation-induced graft copolymerization (Scheme 1B,C). The sample mixture of DC-WCS and PEGMA monomer was exposed to gamma irradiation to create the grafted PEGMA brushes on DC-WCS. When polymer solution is subjected to radiation, most of the radiation energy is absorbed by the solvent [48]. The radiolysis of the solvent initially takes places. Water radiolysis species, such as aqueous electron (e_aq_^−^), hydroxyl (OH^•^), and hydrogen radicals (H^•^), are mainly generated (Scheme 1B). In the case of water/ethanol co-solvent, the radiolysis species of aqueous ethanol is generated (H^•^, ^•^CH_2_CH_2_OH, CH_3_^•^CHOH, and CH_3_CH_2_O^•^) due to C–H and O–H bond cleavage and H-abstraction by the HO^•^ or H^•^ produced from water radiolysis [49]. The HO^•^ and H^•^ reactive species subsequently abstract H atoms at C-1 to C-6 from the polysaccharide backbone of WCS. The reactive macroradicals of WCS are ready to initiate graft-growing chains through an addition reaction with PEGMA monomer. Eventually, the hydrophilic brush shells of comb-like poly(PEGMA) (or PG) were decorated onto DC-WCS to obtain DC-WCS-PG amphiphilic graft copolymers. The DC-WCS-PG building block self-assembled into amphiphilic core–shell NPs. It was supposed that the hydrophobic DC formulated in the inner core of the particles, while the hydrophilic PG brushes favorably located on the surface of NPs (Scheme 1D).

### 3.1. Chemical Structures of DC-WCS-PG

**Figure 1:**
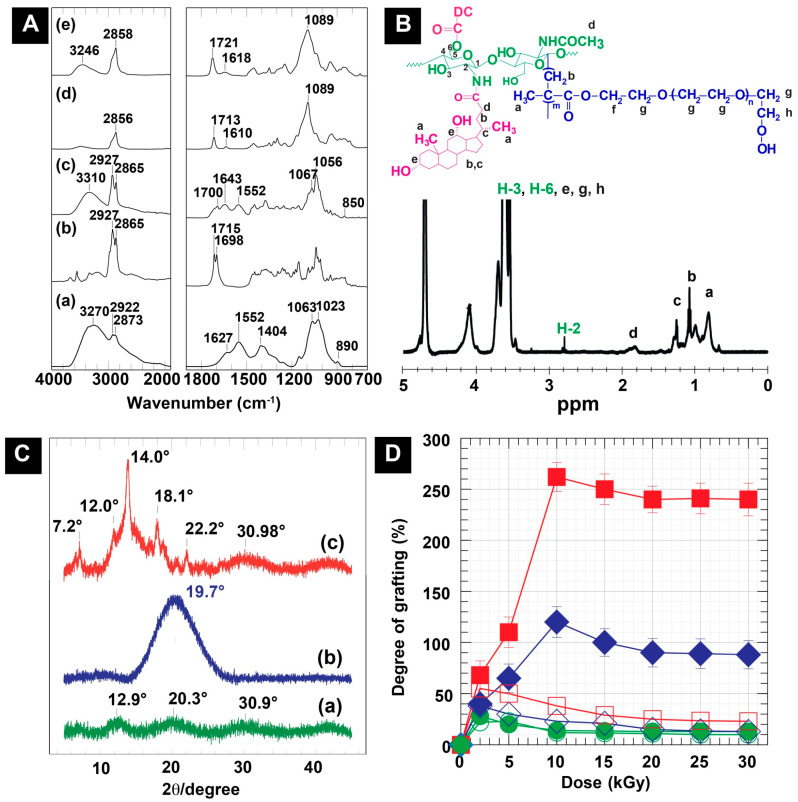
(**A**) FTIR spectra of (**a**) WCS, (**b**) DC, (**c**) DC-WCS, (**d**) PEGMA, and (**e**) DC-WCS-PG. (**B**) ^1^H NMR spectra of DC-WCS-PG. (**C**) XRD patterns of (**a**) WCS, (**b**) DC-WCS, and (**c**) DC-WCS-PG. (**D**) Plots of absorbed doses and the degree of grafting of PEGMA on DC-WCS prepared at different DC-WCS:PEGMA mole ratios of 1:1 (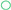), 1:3 (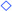), and 1:5 (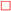) in pure water and 1:1 (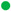), 1:3 (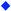), and 1:5 (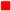) in water/ethanol co-solvent.

To prove the successful synthesis of DC-WCS-PG, the chemical structure based on the functional groups of the sample was clarified using FTIR spectroscopy. The FTIR spectra of WCS, DC, DC-WCS, PEGMA, and DC-WCS-PG are illustrated in Figure 1A. WCS revealed characteristic peaks at 800–1200, 1552, 1627, and 3272 cm^−1^ belonging to the pyranose ring, amide II, amide I, and O–H stretching overlapping with N–H stretching, respectively (Figure 1(Aa)). For comparing DC-WCS (Figure 1(Ac)) with WCS (Figure 1(Aa)), increases in the FTIR peaks at 2864 and 2931 cm^−1^ were found for DC-WCS. These peaks correspond to C–H stretching of the DC structure (Figure 1(Ab)). The appearance of new peaks at 1643 and 1700 cm^−1^ demonstrated the successful conjugation of DC onto WCS through the formation of amide and ester bonds. The FTIR results of DC-CS are as reported in the previous work [38]. 

The reactivity of WCS in comparison with CS for DC conjugation was also assessed using elemental analysis. The chemical composition of DC-WCS was calculated due to an ideal structural conjugation of the DC moieties at the –NH_2_ and –OH groups of WCS. The percentages of C, H, O, and N in the ideal structure were calculated to be 66.48, 9.05, 21.77, and 2.70%, respectively. From the experimental data, the percentages of C, H, O, and N in DC-WCS were found to be 62.17, 8.44, 26.83, and 2.56%. The degree of substitution (DS) of DC on WCS was 98.54%, whereas the DS of DC on CS was 28.12%. It is important to state that the conjugation of DC onto WCS is more efficient than that on CS. This can be explained by the low MW of WCS prepared by radiation-induced chain scission, which increased the reactivity of the obtained WCS due to a higher surface area, shorter chain length, and greater solubility than CS [23]. Therefore, WCS is a promising natural polymer backbone for nano-construction of pharmaceuticals. 

Proving the successful radiation-induced graft copolymerization of PEGMA onto DC-WCS, FTIR spectra were also interpreted, as shown in Figure 1(Ae). The FTIR results were identified on the basis of spectrum analysis and comparison with relevant data reported in the literature [50,51]. Significant absorbance at 2858 cm^−1^ increased in the spectrum of DC-WCS-PG, which is attributed to the symmetric –CH_2_ stretching vibration of the PEGMA structure. The existence of the ester carbonyl (O=C–O–) stretching vibration belonging to the PEGMA structure was found at 1721 cm^−1^. In comparing DC-WSC (Figure 1(Ac)) and PEGMA (Figure 1(Ad)), the ester peak shifted from 1700 and 1713 cm^−1^ to 1721 cm^−1^, implying changes in the new ester environment and vibration, which indicates successful grafting. A strong vibration at 1089 cm^−1^ belonging to the C–O–C stretching in –CH_2_–CH_2_–O– repeating units of the grafted poly(PEGMA) was significantly observed. It can be concluded that the hydrophilic poly(PEGMA) was efficiently grafted onto WCS through the radiation-induced graft copolymerization technique.

To prove the information regarding the molecular structure of DC-WCS-PG, ^1^H NMR was carried out and is shown in Figure 1B. The ^1^H NMR results of DC-WCS-PG were identified on the basis of spectrum analysis and comparison with relevant data reported in the literature [38,39,48,52], and the ^1^H NMR result of DC-CS-PG was in agreement with a previous report [38]. It was found the significant chemical shifts around δ = 0.8–1.3 correspond to the proton resonance of –CH, –CH_2_, and –CH_3_ in the DC structure (assigned to a, b, and c). The chemical shift at 1.6–2.2 ppm was interpreted as proton resonance in the –NHCOCH_2_– of the DC-conjugated WCS (assigned to d). The chemical shift values of H-2 around 2.8 to 3.2 ppm implied a new formation of the amide linkage between the amino (–NH_2_) group of WCS and the carboxylic acid (–COOH) group of DC. The chemical shifts of δ = 3.4–4.1 ppm assigned to H-3, H-6, and –CH_2_–OH (assigned to e, g, and h) were also significantly observed. In addition, a noticeable signal at 4.1 ppm was found and assigned to the protons in the ester bond (–COOCH_2_–, assigned to f). This demonstrated the formation of ester bonds between the –OH of WCS at the C-6 position and the –COOH of DC. The additional peaks and the chemical shifts indicated the successful conjugation of DC onto WCS. For grafting PEGMA onto DC-WCS, the proton resonances of the methane groups in the PEGMA repeating unit (–CH_2_CH_2_O–, assigned to g) appeared at δ = 3.4–4.1 ppm and were located at the same resonance of protons at C-3 to C-6 of CS. Protons resonating at δ = 0.7–1.3 and 1.6–2.2 ppm of the –CH_3_ (assigned to a) and –CH_2_ (assigned to b) in the repeating unit of grafted PEGMA were also found. Moreover, we supposed that the protons in the –COOCH_2_ of PEGMA might also appear at 4.1 ppm (assigned to f). The obtained results agree with previous research [53]. 

### 3.2. Change in the Chain Packing and Crystallinity of DC-WCS-PG

To investigate the change in the chain packing structure of WCS after modification, the XRD patterns of WCS, DC-WCS, and DC-WCS-PG were applied to support the modification (Figure 1C). The broad diffraction peaks at 12.9° and 20.3° (2θ) were assigned to the hydrated crystal and the anhydrous crystals of CS, as reported in previous studies [54,55]. The additional broad diffraction peaks at 30.9° (2θ) may be due to the new crystal structure of WCS. The XRD pattern of WCS (Figure 1(Ca)) showed a broader diffraction pattern due to a more amorphous structure when compared to that of CS [35]. This can be explained by WCS losing its packing structure after the radiation-induced degradation process. X-rays were scattered from the low packing structure or amorphous polymer in many directions, leading to a large bump distributed in a wide range that resulted in low intensity and a broad spectrum instead of high intensity and narrower peaks. 

The XRD pattern of DC-WCS showed a significant broad diffraction peak at 19.7° (2θ) because of changes in the morphology of WCS resulting from insertion of the DC moieties (Figure 1(Cb)). The DC conjugate brought about a more amorphous structure of CS because the hydrophobic bulky structure of DC obstructed the inter- and intra-molecular hydrogel bonding of CS [25,27]. New hydrogen bonds could also occur between the –OH group of DC and the functional groups of WCS (i.e., –NH_2_, –OH, C–O–C, and C=O). In addition, the hydrophobic–hydrophobic interaction of DC moieties on WCS possibly created new crystals in the amorphous structure. In previous studies, the XRD diffraction patterns of polylactic acid (PLA), a hydrophobic polymer, and a grafted CS copolymer also revealed weak and broader diffraction peaks around the 10°–30° (2θ) region, implying that the crystallization of CS was suppressed [56]. 

For PEGMA grafted onto DC-WCS (Figure 1(Cc)), diffraction peaks at 7.2°, 12.0°, 14.0°, 18.1°, 22.2°, and 30.98° (2θ) were observed. The narrow peaks with high intensity indicated the characteristic diffraction of the grafted PEGMA crystals. The peaks at 14.0° and 30.98° (2θ) were assigned to the new crystalline of PEGMA. This phenomenon from diffraction patterns of DC-WCS-PG also agrees with the results for DC-CS-PG in a previous report. For example, the characteristic crystalline peak of PEGMA was found at 14.5° and 30.4° (2θ) when PEGMA was grafted onto CS [27]. As in the previous work, DC-CS-PG was used for comparative study, and diffraction peaks were observed at 12.8°, 18.7°, 21.6°, and 23.9° (2θ) [25,38]. For CS modified with PEG, two strong crystalline peaks at 19.3–23.6° (2θ) and two broad crystalline peaks around 26−31° (2θ) were assigned to the PEG crystalline [55,57]. The change in the XRD pattern reflects the change in the morphological characteristics of PG-WSC-DC when compared with WCS and DC-WCS. Although the XRD pattern of DC-WCS-PGC corresponds to some previous reports, the somewhat different XRD pattern in our observation is likely due to the influence of DC moieties and the WCS backbone. 

### 3.3. Degree of Grafting of PG onto WCS

Figure 1D shows the effect of pure water and water/ethanol co-solvent on the radiation-induced grafting of PG onto WCS under different PG concentrations or mole ratios (WCS:PG = 1:1, 1:3, and 1:5) and different absorbed doses (0–30 kGy). Compared with pure water, the degree of grafting (DG) of PG on WCS in water/ethanol co-solvent was significantly higher. The greater DG suggests that ethanol has a lower polarity index (4.2) than water (10.2). In pure water, DC was suspected to self-assemble as a core compartment, while WCS was covered as a layer on NPs (Scheme 1(Ca)). In the aqueous ethanol solution, the DC moieties promoted extended chains of the hydrophobic DC-WCS because DC is more soluble in ethanol (Scheme 1(Cb)). Therefore, grafting PG easily attached onto WCS of the extended chain. It has been revealed that the solution is one of the parameters for controlling radiation-induced grafting [58]. In a good solvent, the monomer molecules easily shift to the swollen polymer chain and react with the reactive sites on grafted chains, leading to increased grafting efficiency. On the other hand, DC-WCS is unfavorable in water. For this reason, the interaction between DC-WCS and the solvent was not strong enough to overcome the polymer-polymer interaction. The hydrophobic DC moiety escaped from water and self-assembled into the core compartment. This led to the agglomeration of DC-WCS in pure water, and the grafting system was likely performed under heterogeneous reaction. The grafting reaction was limited only on the WCS chain located on the surface of DC-WCS NPs (Scheme 1(Ca)). Hence, the PEGMA monomers tended to form poly(PEGMA) homopolymers instead of graft copolymers with the DC-WCS. 

The relative plots in Figure 1D reveal that monomer concentration influenced the DG. By increasing the feed monomers, the DG is basically increased. By considering water/ethanol co-solvent, increasing PEGMA mole ratio with DC-WCS from 1:1 (0.15% *w/v*) to 1:3 (0.445% *w/v*) and 1:5 (0.75% *w/v*), the DG considerably increased from 28% to 125% and 252%. The DG values were found to be very low in pure water at 2 kGy, and they were 22%, 38%, and 55% for 1:1, 1:3, and 1:5, respectively. 

Besides monomer concentration, the number of free radicals on the polymer backbone and grafting solution system are also important factors [51,58]. The higher the irradiation doses, the greater the DG because of several reactive free radicals formed in the grafting system. A significant increase in the DG was observed at the higher monomer feed ratios of 1:3 and 1:5, as well as at higher given doses up to 10 kGy. The results can be described by the balance of the feeding monomer concentration and the amount of the free radicals that mediates the grafting reaction. If the monomer was already consumed at the reactive site, the DG value was not significantly influenced by increasing the active site or dose. When increasing the dose over the value of 10 kGy, the DG tended to level off and became lower than 125% (1:3) and 252% (1:5). A decrease in DG can be described by (i) a lacking monomer feed ratio, (ii) the termination of active free radicals due to high absorbed doses, (iii) the shortening of already grafted poly(PEGMA) by radiation-induced chain scission, and (iv) obstruction of the mobility of monomer in a viscous system. It is important to note that, by using a high PEGMA concentration, gel formation was evidently found at all irradiation doses because the viscous PEGMA solution obstructed the diffusion of PEGMA to the grafted growing chains [38,58]. It can be concluded that the DG depends on the solvent system, monomer feed ratio, and radiation-absorbed dose. The result suggested that DC-WCS (0.025% *w/v*) dispersing in water/ethanol co-solvent (1:1 *v/v*) containing 0.75% *w/v* of PEGMA irradiated at 10 kGy is an effective condition to create robust grafted PEGMA (~225%) for the preparation of DC-WCS-PG building blocks.

### 3.4. Nanostructured Morphology of DC-WCS-PG

**Figure 2:**
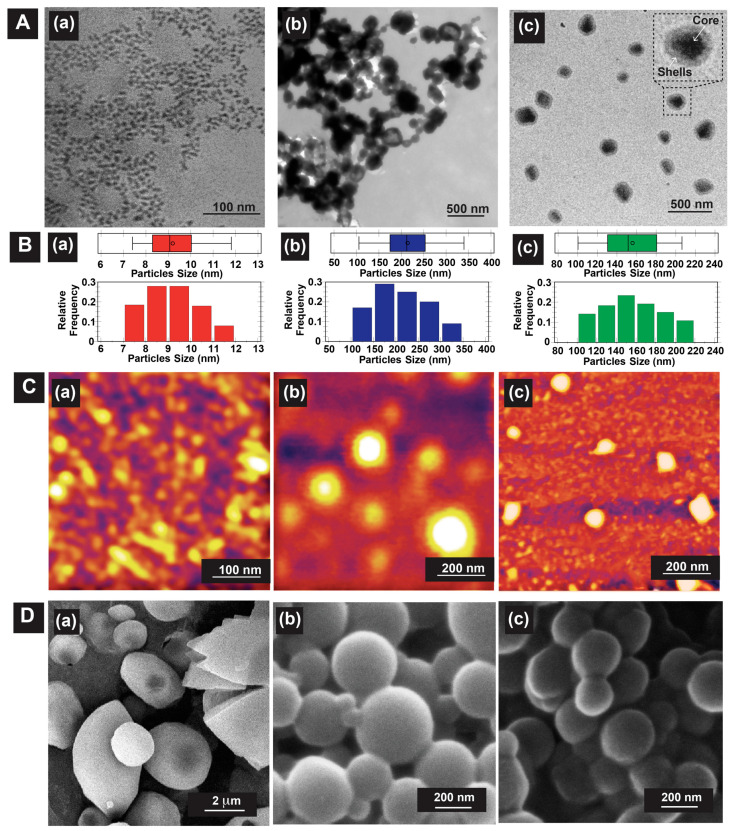
Representative morphological information from (**A**) TEM and (**B**) particle distribution as determined from TEM images, (**C**) AFM, and (**D**) SEM images of (**a**) WCS, (**b**) DC-WCS, and (**c**) DC-WCS-PG (WCS:PEGMA mole ratio = 1:5, co-solvent, 10 kGy, DG = 252%).

To prove our hypothesis for the self-assembly approach of DC-WCS-PG grafted copolymer building blocks, the nanostructured morphology was observed by TEM, SEM, and AFM (Figure 2). The samples were dispersed in water to formulate self-assembling DC-WCS-PG NPs before morphology characterization. Figure 2A shows TEM images of WCS, DC-WCS, and DC-WCS-PG. The particle distribution and boxplot determined from 200 particles (95% confidence with ± 7% precision) in TEM images are shown in Figure 2B. The boxplot demonstrated no outer or anomalous data located outside the whiskers. 

WCS exhibited fine particles with particle sizes of 9 ± 1 nm (Figure 2(Aa)), and it could be found in an agglomerated form with a size ~50 nm [35]. The DC-WCS evidently exhibited condensed and agglomerated spherical particles (Figure 2(Ab)). The particle size of DC-WCS was 214 ± 58 nm. The particle size of DC-WCS was larger than that of WCS. This is due to bulky side groups of DC moieties with a high DS of ~98% on the WCS chains. In water, the hydrophobic DC self-assembled into the core, covering it with WCS polymer chains. For DC-WCS-PG building blocks (DG ~252%), two-phase architectures comprising of a core (dark matter) and shells (grey matter) were clearly observed (Figure 2(Ac)). The grafted poly(PEGMA) shells covering the outer surface had an average thickness of 29 ± 8 nm. Meanwhile, the conjugated DC moieties on WCS were satisfactorily packed as a hydrophobic core. The average core diameter of DC-WCS was 99 ± 18 nm. The average particle size of DC-WCS-PG NPs was 159 ± 29 nm. Compared with DC-WCS, the average particle size of DC-WCS-PG NPs was smaller because each component in the water system after drying was well packed. 

AFM images were used to confirm particle morphology (Figure 2C). Similarly, the WCS NPs revealed fine and agglomerated particles with a size of 35 ± 13 nm (Figure 2(Ca)). The DC-WSC NPs showed spherical morphology with a size of 220 ± 27 nm (Figure 2(Cb)). In the case of PG-WSC-DC NPs (Figure 2(Cc)), distinguished particles with a spherical shape were obviously observed. The images observed by AFM support the morphology of PG-WSC-DC NPs observed by TEM. The particle size of DC-WCS-PG NPs was 91 ± 9 nm. 

Particle morphologies of the lyophilized sample powders were also observed by SEM. Large spherical particles with irregular shapes were found (Figure 2(Da)). The hydrogen bond interaction of non-chemically modified WCS led to close-packed chains forming largely agglomerated particles (~2.9 ± 0.6 μm). The surface morphology of DC-WCS revealed clear and distinguished spherical particles with quite smooth particle surfaces (Figure 2(Db)). The DC moiety on the conjugated WCS plays an important role in controlling the morphology of the self-assembled NPs. The particle size of DC-WCS was ~299 ± 48 nm. Similarly, DC-WCS-PG (Figure 2(Dc)) also displayed spherical particles with a size of ~268 ± 38 nm, which was smaller than DC-WCS. 

The hydrodynamic size (D_H_) of NPs in aqueous solution was also characterized using dynamic light scattering (DLS) measurements. The D_H_ and zeta potential values of WCS, DC-WCS, and DC-WCS-PG were 81 ± 16 nm (2.50 mV), 226 ± 12 (2.28 mV), and 372 ± 15 nm (−3.60 mV), respectively. Undoubtedly, the D_H_ of the DC-WCS-PG NPs was larger than that of the DC-WCS NPs because the PEGMA-grafted shells expanded in the aqueous solution. The nano-morphological information observed by TEM, AFM, and SEM, including the D_H_ from DLS, was consistent. The results confirm the successful formation of self-assembling NPs from the DC-WCS-PG building blocks. It is clear that tailoring the chemical structure of WCS led to control of its nanoarchitecture as a result of the interaction of each component in the polymeric building block and the given solutions. 

### 3.5. Water Solubility

**Figure 3:**
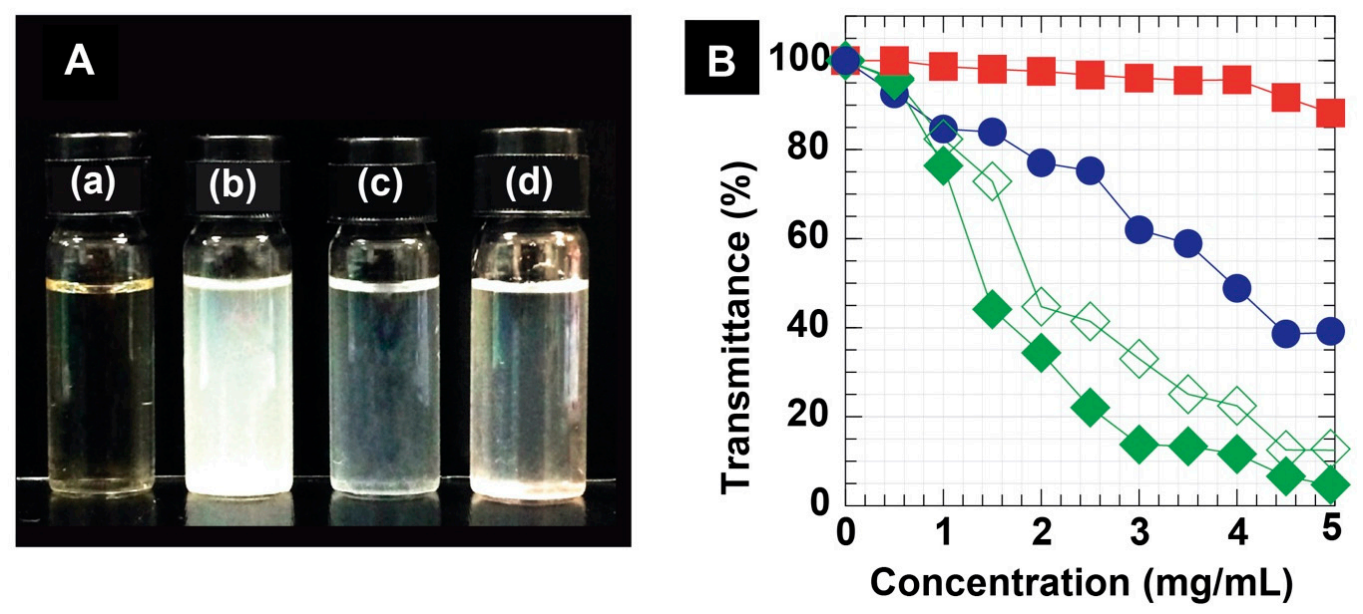
(**A**) Physical appearance of (**a**) WCS, (**b**) DC-WCS in water, (**c**) DC-WCS in ethanol, and (**d**) DC-WCS-PG in water at 1.5 mg/mL. (**B**) Transmittance (%) at a wavelength of 600 nm for WCS (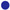), DC-WCS in water (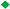), DC-WCS in ethanol (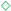), and DC-WCS-PG (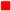).

The physical appearances of representative sample solutions at 1.5 mg/mL are shown in Figure 3A. The WSC NPs sample was light-yellow and transparent (Figure 3(Aa)). The DC-WCS NPs solution was turbid, implying insolubility in water (Figure 3(Ab)) due to DC hydrophobic moieties. However, the DC-WCS NPs exhibited more solubility in ethanol, as seen in the transparent solution (Figure 3(Ac)). With robust grafted comb-like PEGMA shells, water solubility and the dispersion of the DC-WCS-PG NPs were significantly improved (Figure 3(Ad)). 

The water solubility/dispersion of DC-WCS-PG NPs was observed using a UV spectrophotometer. The percentage of transmittance at a certain visible wavelength of 600 nm was measured and plotted as a function of NP concentrations (Figure 3B). The higher percent transmittance, the greater solubility of the sample. By increasing the concentrations of samples, the percent transmittance was generally reduced. In the case of WCS, the percent transmittance gradually reduced from 100% to ~40% when the concentration increased from 0.02 to 5 mg/mL. WCS exhibits good water dispersion and shows transmittance of above 80% at concentrations as high as 5 mg/mL. The result confirms that WCS and PEGMA supported the water solubility and dispersion of DC-WCS-PG in a neutral pH at room temperature.

The percent transmittance of DC-WCS in water significantly reduced from 100 to 4.4%. DC-WCS in ethanol exhibited somewhat higher percent transmittance, implying greater solubility. The results clearly demonstrated the effect of hydrophobicity due to DC moieties on the solubility property. DC-WCS-PG exhibited outstanding dispersion in water, providing a percent transmittance at 600 nm of above 80% in a wide range of concentrations (0–5.0 mg/mL). The result suggests that both WCS and PEGMA shells play an important role in enhancing the water dispersion of DC-WCS-PG amphiphilic NPs. 

### 3.6. Drug Encapsulation Efficiency

PTX is a nonionic molecule with high lipophilicity and is one of the most successful anticancer drugs discovered in the past few decades. BBR is a potent alkaloid hydrophobic drug with a positive charge and is also considered as a promising natural product for the treatment of metabolic diseases. Similar to the other anticancer or antibiotic compounds, both PTX and BBR are poorly soluble in aqueous media and are not applicable for conventional aqueous injection solutions [2,53]. Enhancing the solubility of water-insoluble drugs using amphiphilic core–shell NPs is a way to improve drug formulations [59] and drug delivery in the biological system. In this section, the abilities of newly developed DC-WCS-PG NPs as a potential drug nanocarrier were evaluated. The drug encapsulation efficiency (EE) and loading content (LC) of the DC-WCS-PG NPs (having a low MW of WCS) prepared in this work were measured in comparison with the DC-CS-PG NPs (having a high MW of CS) of a previous study (ref). EE was calculated from the available drug amount loaded into the NPs in comparison with an initially added drug amount. LC was calculated as the mass ratio of drugs to the mass of nanomedicines. 

**Figure 4:**
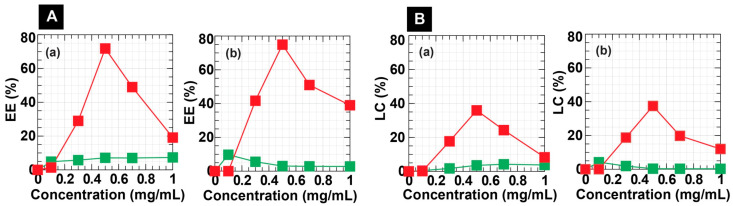
(**A**) Drug encapsulation efficiency (EE) and (**B**) loading content (LC) of (**a**) PTX and (**b**) BBR in DC-CS-PG (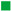) and DC-WCS-PG (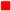) NPs.

Figure 4A,B reveal plots of the EE and LC of DC-WCS-PG NPs against drug concentrations. The EE and LC showed a similar tendency to drug concentrations. Undoubtedly, LC should be high when the NPs have a lower EE at a higher drug concentration of >0.5 mg/mL. In the BBR and PTX concentration ranges of 0–1.0 mg/mL, the EEs of DC-CS-PG NPs were quite low (around <10%). The EE of DC-WCS-PG NPs was significantly higher than that of DC-CS-PG NPs prepared from high-MW CS. The greater EE of DC-WCS-PG is attributed to the alteration of WCS. The DC on WCS was 3.5-fold higher than that on CS. High moeities of DC on WCS provided a larger area for hydrophobic drug interaction and encapsulation. 

For DC-WCS-PG NPs, EE and LC tended to increase when increasing PTX and BBR concentrations to 0.3 and 0.5 mg/mL. In this range of concentration, the rate of diffusion of drugs across the surface compartment of the NPs is still proportional to the concentration gradient. Based on Fick’s laws of diffusion, the drug basically diffused from a higher concentration of the drug outside NPs to the lower concentration of the drug inside the NPs. At drug concentrations over 0.5 mg/mL, both EE and LC continuously declined. This is due to the capacity of the DC-WCS-PG NPs to encapsulate a certain amount of PTX and BBR. The excessive amount of drug molecules could not diffuse and occupy the NPs because the NPs had no more space and lacked binding sites. It was also suggested that not only the diffusion control process but also the mobility of the drug influences drug encapsulation efficacy at higher drug concentrations. 

Considering PTX at 0.5 mg/mL, the maximum PTX-EE of DC-WCS-PG (159 ± 29 nm) was 71.84 ± 0.28% (Figure 4(Ab)), and this is equivalent to the PTX-LC of 35.92 ± 0.14% (ca. 359.2 mg/g) (Figure 4(Bb)). The mode of encapsulation of PTX into the DC-WCS-PG NPs was assumably due to a hydrophobic–hydrophobic interaction between the PTX molecules and the conjugated DC moeties. An example of the PTX-encapsulated liposome has been reported. The PTX-LC of liposome (~100–130 nm) was 4.5 ± 0.1% (ca. 45 mg/g) [60]. The DC-WCS-PG NPs exhibited almost eight-fold higher PTX-LC. This can be considered in terms of the different chemical structures of the NPs. In our case, large molecules of hydrophobic DC bile acid provided a larger space for effective encapsulation of the complex and the bulky structure of the PTX. On the other hand, the aliphatic hydrophobic tails assembled in the liposome core provide small cavities for PTX encapsulation. Accordingly, the self-assembled DC-WCS-PG NPs exhibited higher PTX-LC than the self-assembled liposomes. Likewise, the amphiphilic polymeric micelles of DC and folic acid (FA)-modified CS were also developed in a previous study [61]. The PTX-LC of FA-CS-DC micelles was found to be 9.1% (ca. 91 mg/g). Compared with the EE of liposomes, the EE of FA-CS-DC micelles was two-fold higher. This agrees with our postulation that the DC core of amphiphilic NPs is preferential for PTX encapsulation when compared with the aliphatic core of the liposome. Comparing the DC-WCS-PG NPs with different amphiphilic CS derivatives, it was found that PTX-LC in our case was four-fold higher than FA-CS-DC micelles. This can also be described in terms of the conjugated DC moieties. The DS of DC on FA-CS-DC (15.8%) was six-fold lower than that of DC on DC-WCS-PG NPs (98.54%). This may be the reason for the improved PTX-LC of DC-WCS-PG NPs. Meanwhile, it is also suggested that the hydrophilic poly(PEGMA) brushes could hinder the diffusion and encapsulation efficiencies of the hydrophobic PTX in the DC core. The comparative data explained the influence of the molecular components of the self-assembled building blocks on LC. In summary, the water-soluble amphiphilic DC-WCS-PG NPs displayed higher efficiency in terms of encapsulating the water-insoluble PTX compared to previous studies. 

For BBR encapsulation, the BBR-EE of DC-WCS-PG NPs exhibited a similar phenomenon to PTX-EE. At 0.5 mg/mL, the maximum BBR-EE of DC-WCS-PG NPs was 74.91 ± 2.99% (Figure 4(Aa)), corresponding to BBR-LC of 37.46 ± 1.49% (ca. 374.6 mg/g) (Figure 4(Ba)). For comparison with previous studies, the encapsulation efficiency of BBR into CS microspheres prepared from ionic crosslinked CS with sodium tripolyphosphate (STTP) at a BBR/CSNPs ratio of 3:5 was ~100 mg/g [62]. The LC of DC-WCS-PG NPs observed in our work was ~three-fold higher than CS-STTP NPs. The STTP could help to entrap BBR due to electrostatic interactions between PTTP and BBR; however, the high MW of CS (~9.0 × 10^5^ Da) in CS-STTP microspheres of larger particle size (~10 μm) might obstruct encapsulation efficiency. As in our work, the high MW of CS (~ 3 × 10^5^ Da) used in DC-CS-PG NPs exhibited lower BBR-LC (~50 mg/g), whereas the low MW of WCS (~5000 Da) used in DC-WCS-PG NPs led to 7.5-fold greater LC (Figure 4(Bb)). In addtion, it was also reported that folate acid-modified CSNPs (258.2 ± 9.1 nm) had BBR-LC of 8.17 ± 1.12% (ca. 81.7 mg/g) [63]. For comparative analysis, the BBR-LC of the folate-modified CSNPs was approximately ~4.6-fold lower than DC-WCS-PG NPs. The high efficiency of DC-WCS-PG for BBR encapsulation is suspected to be due to the influence of molecular size, the hydrophobic core, and the building block components, i.e., WCS chains, DC moeties, and poly(PEGMA) brushes. BBR is a positively charged and hydrophobic drug; therefore, sufficient DC moeties in the NP core provided a large space for BBR encapsulation. Furthermore, oxygen- and nitrogen-rich poly(PEGMA) brushes and WCS chains result in negative charges that promote stronger interactions with the postively charged BBR. 

It is also important to note that the DC-WCS-PG NPs have higher efficiency encapsulating BBR than PTX. The PTX drug is more hydrophobic without charge, whereas the BBR drug contains a positive charge at the nitrogen atom. Therefore, the hydrophobic PTX may favor locations inside the hydrophobic DC core of the DC-WCS-PG NPs. The encapsulation mode of the PTX in the DC-WCS-PG NPs is supposed to only be a hydrophobic–hydrophobic interaction (Scheme 1E). Since BBR has both hydrophobicity and a positive charge, it is not only possibly encapsulated into the DC core, but is also possibly stabilized within the WCS chains and the poly(PEGMA) brushes (Scheme 1E). The electrostatic interaction governs BBR’s association with WCS and poly(PEGMA) because BBR and NP components carry permanent charges. This agrees with an increase in zeta potential from the negatively charged DC-WCS-PG NPs (−3.60 mV) to the positively charged BBR-loaded DC-WCS-PG NPs (4.58 mV). As in previous studies, BBR can be entrapped into the nanocarrier in various ways, such as (i) hydrophobic–hydrophobic interactions, (ii) simple electrostatic interactions (e.g., with liposome, chitosan, PVA), and (iii) physisorption inside mesoporous silica NPs and metallic NPs [45,63,64]. The achieved results indicate that drug structure, solubility, concentration, and copolymer building blocks with different structures (e.g., hydrophobic/hydrophilic moeties, MW, and charge of polymer) influence the EE of the amphiphilic core–shell DC-WCS-PG NPs.

### 3.7. In Vitro Controlled Release of Drugs

**Figure 5:**
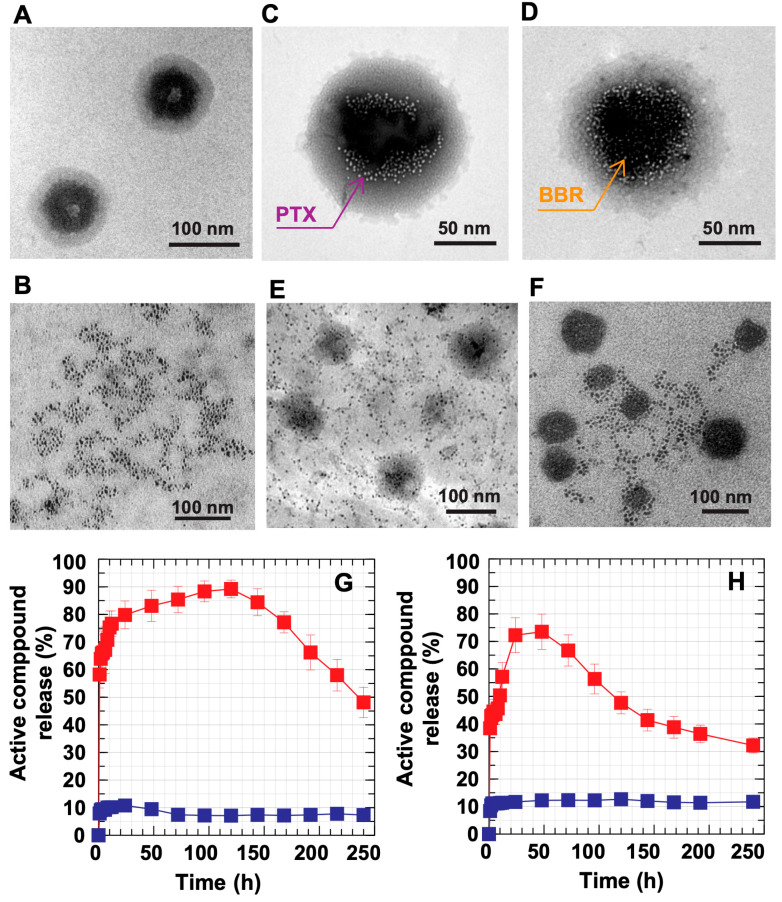
Controlled drug release behavior of DC-WCS-PG NPs. Representative TEM images of (**A**) blank DC-WCS-PG NPs, (**B**) pure PTX, (**C**) BBR-encapsulated DC-WCS-PG NPs, and (**D**) PTX-encapsulated DC-WCS-PG NPs; (**E**) BBR releasing from DC-WCS-PG NPs in PBS buffer (pH 7.4) at 48 h; (**F**) as in (**E**) but for PTX. Kinetics release profile of (**G**) PTX and (**H**) BBR from DC-WCS-PG NPs (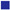) in PBS buffer (pH 7.4) compared with free drug (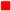).

To observe the controlled release function of the DC-WCS-PG NPs, the release of both BBR- and PTX-loaded DC-WCS-PG NPs was performed using the dynamic dialysis method in PBS buffer at a pH of 6.5 and 7.4 within a period of 240 h. The in vitro controlled release experiment was designed in a circulatory system to mimic the intravenous injection of a drug [65]. Figure 5A shows the core–shell architecture of the DC-WCS-PG NPs without drug loading. The representative morphology of the free PTX is shown in Figure 5B. The PTX- and BBR-loaded DC-WCS-PG NPs are shown in Figure 5C,D. It is suspected that the PTX and BBR appeared in small spots inside the NPs. PTX was significantly located along the boundary of the inner core. It was demonstrated that the water-insoluble PTX could be incorporated with the DC conjugates on the WCS backbone. Similarly, BBR was encapsulated into the NP core and interacted with or was entrapped by the WCS chains through electrostatic interactions. The evidence from TEM images agrees with our expectations and is illustrated in Scheme 1E. The morphological data supported the encapsulation performance of the DC-WCS-PG amphiphilic core–shell NPs. Figure 5E,F show the representative morphologies of PTX and BBR releasing from the DC-WCS-PG NPs in PBS buffer at a pH of 7.4 after 48 h. Tiny particles similar to those in Figure 5B spread in the media phase around the DC-WCS-PG NPs, implying that PTX and BBR were released from the DC-WCS-PG NPs.

The time-dependent in vitro PTX and BBR release profiles are shown in Figure 5G,H. In the simulated circulatory system, the unloaded drug (blank symbol) exhibited a time-dependent profile. At an initial stage, the unloaded drug showed a robust release of upto ~80% when time was increased from 0 to 24 h. This is due to the diffusion-controlled process, in which the drug diffused from a higher concentration across the membrane to the lower one. Within the period of 24–120 h, PTX slowly released and increased from ~80% to a maximum amount of ~90%, mostly because of the balance between concentrations inside the membrane and outside the environment (Figure 5G). The changes in the release rate of free PTX within 24–120 h might be due to parameters besides concentration controlling the diffusion process. The cause is unclear, though it is assumed to be influenced by several factors, such as the hydrophobicity of the drug molecule, changes in the drug’s molecular size due to aggregation, interactions between drug and membrane, and changes in membrane pore size due to prior release and interaction. After 120–240 h, the release of free PTX gradually declined from 90% to ~45% because the remaining amount of PTX inside the membrane dramatically decreased. 

Similary, the unloaded BBR was also dramatically released by up to ~70% within a period of 24 h (Figure 5H). The release of BBR also decreased from 70% to 30% within the period of 48–240 h because the amount of BBR significantly decreased within the initial 24 h. During the first stage, there was a phenomenon of drug delivery at high release rates, which is called a “burst release phase” [66]. This burst release causes drug side effects or unnecessary drug therapy, which is one of drug therapy’s main problems [67]. The controlled release process can be applied to solve this problem in order to reduce the amount of unnecessary drug used in patient therapy. In our studies, the robust release behavior of free PTX and BBR at the initial time of 0–24 h affirmed that the free drug exhibits a non-controlled release function. It is clear that when the free drug is administrated into the biological system, the drug robustly spreads without effective utilization, thus leading to some adverse effects due to the excessive dose. As has been reviewed in the literature [66], drug delivery systems can be summarized into four categories: monophasic [68], burst biphasic [69], delayed biphasic [70], and triphasic [71]. In our experiment, it can be demonstrated that free PTX and BBR exhibited the “burst biphasic” phenomenon of drug release according to the previous review. 

For drug-loaded DC-WCS-PG NPs at pH 7.4 (filled symbol), it was found that both PTX (Figure 5G) and BBR (Figure 5H) were significantly released by up to approximately 10–11% within the initial period of 0–4 h. In this stage, the “burst release phase” was found, where the drug was typically rapidly released upon initial exposure of the drug-loaded DC-WCS-PG NPs to the aqueous environment. The result corresponds to the previous observation in the case of PLGA microparticles [71]. Both PTX- and BBR-loaded DC-WCS-PG NPs exhibited a constant release profile of ~10% within the period of 4–240 h. The release profiles showed a steady-state drug concentration in the circulation system after logistic growth during the first stage. For drug-controlled release, the steady state can be applied to maintain the drug within the therapeutic period and control the drug concentration so that it does not exceed the threshold of toxicity [66]. The result suggests that the DC-WCS-PG NPs meet the ideal drug-controlled release function. The persistent slow release of PTX and BBR from the NPs affirms the applicablity of DC-WCS-PG NPs in drug-controlled release applications. 

The behavior of drug-controlled release from polymer matrices normally depends on MW, dissolution, diffusion, osmosis, partitioning, swelling, and erosion [72]. In our case, the amphiphilic DC-WCS-PG NPs consisting of different components displayed different properties. For example, DC exhibits hydrophobicity, poly(PEGMA) brushes are rich in oxygen atoms, hydroxyl groups provide hydrophilic characteristics, and WCS as a cationic provides a pH-responsive function. 

**Figure 6:**
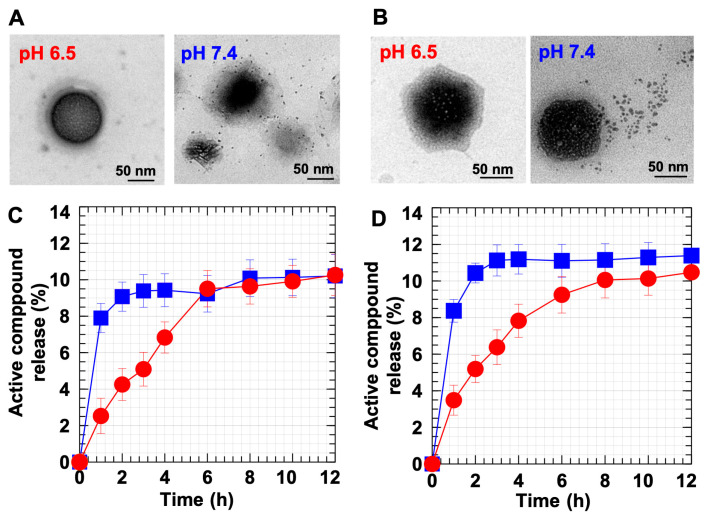
Controlled drug release behavior of DC-WCS-PG NPs in PBS buffer at different pHs. Representative TEM images of (**A**) PTX and (**B**) BBR releasing from DC-WCS-PG NPs in pH 6.5 and pH 7.4 buffer solution at 1 h. Kinetics release profile of (**C**) PTX and (**D**) BBR from DC-WCS-PG NPs in PBS buffer at pHs of 6.5 (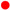) and 7.4 (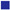).

To clarify the pH-responsive function of the DC-WCS-PG NPs, PTX and BBR release behaviors from DC-WCS-PG NPs under different pHs were assessed. Figure 6A shows the morphology of PTX releasing from DC-WCS-PG NPs at a pH of 6.5 and 7.4 after 1 h. At pH 6.5, WCS was protonated, and the WCS chains expanded as suspected (shown by the dark-field area). Consequently, most of the PTX was still inside the NPs and had only been slightly released at 1 h. At pH 7.4 on the other hand, the WCS was in a deprotonate form; thus, WCS chains were assumably contracted in the core in the dark-field area. Subsequently, PTX was released from the NPs. These phenomena resemble those illustrated in Scheme 1F. Compared to PTX, similar phenomana were also observed for BBR release behavior at a pH of 6.5 and 7.4 (Figure 6B). Different morphologies are reasonable due to different compartments and different interactions between drug molecules and NP components. 

Figure 6C,D show the time-dependent release profiles of PTX and BBR, respectively. PTX showed the logistic growth profile of the burst release phase during the first period of ~4 h (for pH 7.4) and ~8 h (for pH 6.4) (Figure 6C). After the logistic growth, the release profile moved towards a steady rate of ~10% drug release. Similarly, for BBR (Figure 6D), logistic growth was also found within the time periods of ~4 h (at pH 7.4) and ~8 h (at pH 6.5). At pH 7.4, the maxium BBR release was ~11%, and BBR release was maintained at a steady rate after 4 h. This phenomenon agrees with the monophasic drug release profile suggested in a previous review [66]. The monophasic profile is a desirable form of drug release kinetics because in vitro drug concentration in media circulation (or in the body) is completely predictable throughout the time course. Under pH 7.4 (pH > pKa of CS), CS preferred to be contracted to chains, leading to shrinkage of the NPs and smaller particle sizes. The TEM images also display that both PTX and BBR were pressed out and released from the NPs to the environment (Figure 6A,B). 

Compared to pH 7.4, maximum BBR release at pH 6.5 was ~10%, and BBR release reached a steady state at ~8–12 h. Under pH 6.5, the longer time to reach steady-state release implies transformation of DC-WCS-PG NPs due to the protonation of CS. Under acidic conditions, the –NH_2_ of CS can be protonated to –NH_3_^+^, leading to chain expansion due to repulsive force. When the DC-WCS-PG NPs expanded, the drug molecules were confined in the expanded polymer chains of the NPs (pH 6.5) (Figure 6B and Scheme 1F). This led to more time being required to reach a steady state under pH 6.5 because the drug molecules were covered internally by NPs. The pH-responsive controlled release phenomon of DC-WCS-PG NPs owing to CS function agrees with previous reports. Tamoxifen was more rapidly released from the CS-based NPs at pH 4.0 and 6.0 than at pH 7.4 [73]. In addition, it is also worth noting that the pH-responsive function of CS is suitable for oral drug delivery because the drug would not be released or destroyed in the stomach, where the pH is ~1.5–3.5 [73,74]. In normal blood at 37 °C, the pH is 7.4 [75]. In addition, normal cells have an extracellular pH value of around 7.4, though the pH of cancer cells can be typically lower, varying from 6.7–7.1 [76]. Thus, information regarding the pH response of DC-WCS-PG NPs in our observation supports the beneficial phenomenon of drug-controlled release in blood, as well as in normal and cancer cell environments. 

### 3.8. Antimicrobial Behavior of BBR-Loaded DC-WCS-PG NPs against S. ampelinum

BBR is considered as one of the most promising natural products for the treatment of metabolic diseases because of its pharmacological activities, such as antimicrobial activity and anti-inflammatory properties. In terms of plant disease, *Sphaceloma ampelinum de Bary (S. ampelinum)* is a cause of anthracnose or scab disease in grapevine, which is one of the most serious fungal diseases of grape in tropical areas. In this section, the antifungal activity of BBR against the growth of *S. ampelinum* was assessed. Zones of inhibition were applied to measure antibiotic resistance activities. At 14 days, the inhibition zone increased to 0.00, 0.52, 1.18, 2.08, and 2.08 cm when the concentration of the BBR-loaded DC-WCS-PG NPs was increased to 10, 100, 1000, 10,000, and 100,000 ppm, respectively. To determine the controlling release function of NPs, free BBR and BBR-loaded DC-WCS-PG NPs were applied as an antimicrobial agent in the treatment of *S. ampelinum.* Accumulative BBR release leading to the enhanced inhibition of *S. ampelinum* was assessed though inhibition zone diameter. An inhibition zone was not found for neat DC-WCS-PG NPs. 

**Figure 7:**
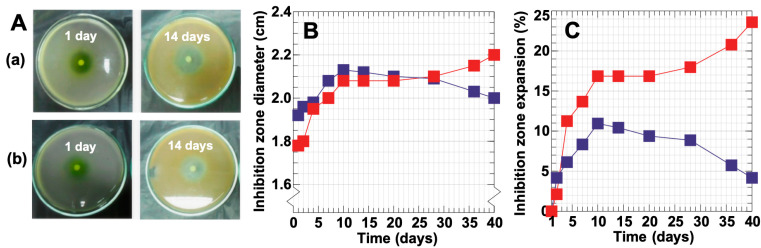
(**A**) Photograph of the antifungal efficiency of (**a**) free BBR and (**b**) BBR-loaded DC-WCS-PG NPs. (**B**) Inhibition zone diameters and (**C**) inhibition zone expansion of free BBR (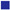) and BBR-loaded DC-WCS-PG NPs (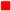) against *S. ampelinum*.

Figure 7A contains representative photographs of inhibition zones after treatment with free BBR and BBR-loaded DC-WCS-PG NPs at day 1 and after 14 days. The inhibition zone diameter of BBR at day 1 was 1.92 ± 0.10 cm, and that of BBR-loaded DC-WCS-PG NPs was 1.78 ± 0.12 cm (Figure 7B). At 14 days, the inhibition zone diameters of BBR and BBR-loaded NPs increased to 2.10 ± 0.09 (9.38%) cm and 2.08 ± 0.14 cm (16.85%), respectively. The inhibition zone diameter of BBR was slightly greater than that of BBR-loaded NPs because BBR freely and readily inhibited fungal growth, whereas the BBR-loaded NPs slowly released and gradually inhibited fungal growth. For a longer period of up to 35–40 days, the BBR-loaded NPs showed their potential to inhibit fungal growth to a greater extent than free BBR. This phenomenon suggests the slow release efficiency of BBR-loaded NPs.

To observe changes in the inhibition zone compared to the initial time (1 day), the relative percentage of inhibition zone expansion was calculated and plotted against incubation time (Figure 7C). With increasing time, the percentage of inhibition for BBR-loaded NPs tended to increase during the initial stage and then leveled off, decreased, or eventually increased after a certain period. For free BBR, the percentage of inhibition increased from 0 to 11% after treatment for 0–10 days. Beyond 10 days, the percentage of inhibition continuously declined from 11% to 4%. Free BBR robustly inhibited the growth of *S. ampelinum* within the initial period and then leveled off. The increased inhibition of free BBR was due to the burst release of free BBR from the surface to the surrounding system. In the case of the BBR-loaded DC-WCS-PG NPs, the percentage of inhibition also robustly increased from 0 to 17% when incubating for 0–10 days. This phenomenon corresponds to the burst release phase previously described. The BBR-loaded DC-WCS-PG NPs could retain 17–18% inhibition within the period of 10–30 days. It is also important to note that the BBR-loaded DC-WCS-PG NPs showed an increase in the percentage of inhibition of up to almost 25% at 30–40 days. It has been reported that the properties of NPs (such as particle size/porosity, polymer molecular weight, and drug hydrophobicity/hydrophilicity) influence the burst release behaviors of PLGA microparticles [66]. In our case, the BBR molecules were possibly loaded not only into the DC core of NPs, but also into the main constructed WCS polymer chains, as well as the grafted poly(PEGMA) shells of the DC-WCS-PG NPs. Accordingly, the BBR trapped nearby the surface of the DC-WCS-PG NPs initially inhibited the growth of *S. ampelinum*, as indicated by the burst release profile. Meanwhile, the BBR encapsulated inside the NPs gradually diffused out to the surface of the NPs and was eventually released to the surrounding area. The kinetics profile of the percentage of inhibition implies that the drug-controlled release function of the DC-WCS-PG NPs can prolong BBR drug efficiency. 

### 3.9. Cytotoxicity of PTX-Loaded DC-WCS-PG NPs towards Breast Cancer and Human Skin Fibroblast Cells

**Figure 8:**
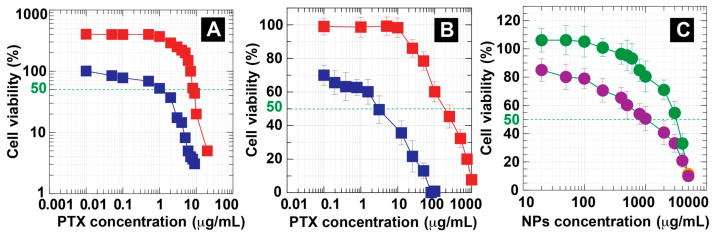
Cellular viabilities of (**A**) breast cancer cells and (**B**) fibroblast cells after treatment with free PTX (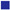) and PTX-loaded DC-WCS-PG NPs (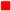). (**C**) Cellular viabilities of MCF-7 breast cancer cell lines (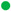) and the CRL 2522 fibroblast cell line (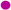) after treatment with the blank DC-WCS-PG NPs.

PTX has been approved by the US FDA for the treatment of breast, ovarian, and non-small-cell lung cancer [1,77]. In this work, the cytotoxicity of PTX in comparison with PTX-loaded DC-WCS-PG NPs against CRL 2522 fibroblasts and MCF-7 breast cancer cells was examined using the MTT assay. This experiment was performed using a wide range of PTX concentrations (0–100 μg/mL) and the cells were incubated for 24 h before measurement. Figure 8A shows the cell viability of breast cancer cells against PTX concentration. The breast cancer cells presented a dose-dependent reduction in cell viability after contact with free PTX and the PTX-loaded DC-WCS-PG NPs. The PTX-loaded DC-WCS-PG NPs showed higher cell viability than free PTX. At a PTX concentration of 0–1 μg/mL, the cell viability of PTX-loaded DC-WCS-PG NPs did not significantly change, while that of free PTX gradually decreased. Cell viability was dramatically reduced when PTX concentration was greater than 1 μg/mL. 

Half maximal inhibitory concentration (IC_50_) is a value that indicates the ability of a drug to inhibit a specific biological activity. The lower the value of IC_50_, the higher the inhibition ability. The PTX-loaded DC-WCS-PG NPs and unloaded PTX displayed IC_50_ values of 8.31 ± 0.71 μg/mL and 1.17 ± 0.64 μg/mL, respectively. The IC_50_ of PTX-loaded DC-WCS-PG NPs was seven-fold higher than unloaded PTX. Our results, therefore, demonstrate that DC-WCS-PG NPs play a significant role in slowing drug release. As in previous studies, IC_50_ was not observed for PTX-loaded nanostructured lipid carriers (NLCs) treated with MDA-MB-231 breast cells within the concentration range of 0–2500 nM (or ~2.13 μg/mL) [78]. At the highest concentration (2500 nm), cell viability was found to be 56.0 ± 3.2%. Considering the same concentration as the previous report, PTX-loaded DC-WCS-PG NPs displayed cell proliferation of MCF-7 breast cancer cells lines of about 285 ± 28%. The breast cancer cell viability of PTX-loaded DC-WCS-PG NPs was eight-fold higher than that of unloaded PTX (35.07 ± 14%). In terms of previous studies, breast cancer cell viability after treatment with the PTX-loaded lipid-based nanocarrier (38.0 ± 5.0%) was only 1.47-fold higher than unloaded PTX (56.0 ± 3.2%). For comparison, the cytotoxicity of PTX-loaded DC-WCS-PG NPs was five-fold lower than the PTX-loaded lipid-based nanocarrier. This can be explained in terms of nanoarchitecture and composition effects. Since the lipid-based nanocarrier is basically unstable compared to the polymer-based nanostructure, the encapsulated drug molecules are easily released and subsequently display treatment efficiency towards breast cancer cells. Although high drug release attributes to a significant effect against cancer cells, the adverse effects of an excessive amount of the drug on non-cancerous cells would also be an important issue to consider for balanced treatment. 

Herein, non-cancerous cells, i.e., fibroblast (L929) cells, were also treated by free PTX and PTX-loaded DC-WCS-PG NPs and were incubated for 24 h for cell viability determinations. The kinetics for cell viability are presented in Figure 8B. As expected, no significant cytotoxic activity was observed for the PTX-loaded DC-WCS-PG NPs at concentrations of 0–10 μg/mL. The IC_50_ of PTX-loaded DC-WCS-PG NPs with fibroblast cells was found to be as high as 203 ± 29 μg/mL. As described, the IC_50_ of PTX-loaded DC-WCS-PG NPs in the treatment of MCF-7 breast cancer cells was only 8.31 μg/mL. It is important to note that the PTX-loaded DC-WCS-PG NPs did not show cytotoxicity to non-cancerous cells at the considered IC_50_ of the cancer cells. For unloaded PTX, the IC_50_ with fibroblast cells was 2.92 ± 0.61 μg/mL. The results concerning the cytotoxicity of unloaded PTX with fibroblast cells in our study agree with the previous literature (IC_50_ ~2.13 μg/mL) [78]. Unloaded PTX exhibited ~70-fold higher toxicity towards fibroblast cells than PTX-loaded DC-WCS-PG NPs. The result demonstrates that the DC-WCS-PG NPs also displayed preferable nanocarrier characteristics for controlled drug release applications in terms of slowing drug release and reducing side effects. 

The cytotoxicity of the DC-WCS-PG NPs without drugs was also evaluated with MCF-7 breast cancer cells and CRL 2522 human skin fibroblast cells (Figure 8C). Both cell lines presented a dose-dependent reduction in cell viability after treatment with the neat DC-WCS-PG NPs. A plot of cell viability against NP concentration demonstrates that the DC-WCS-PG NPs exhibited more toxicity towards breast cancer cells than the human skin fibroblast cells. The IC_50_ of DC-WCS-PG NPs with breast cancer cells and non-cancerous cells was 1237 ± 89 μg/mL and 3214 ± 102 μg/mL, respectively. The overall cell viability results and the IC_50_ values imply that neat DC-WCS-PG NPs have low toxicity. Somewhat higher cell viability of >100% led us to suspect that the metabolism of fibroblast cells was able to adapt and somewhat proliferate under DC-WCS-PG treatment within a low concentration range of 0–100 μg/mL. At concentrations >100 μg/mL, fibroblast cell viability gradually decreased; however, the IC_50_ was still very high. It is a good sign that no significant cytotoxicity was found against non-cancerous cells when DC-WCS-PG NPs were used. 

Compared to fibroblast cells, a significant dose-dependent reduction in breast cancer cells was observed. The neat DC-WCS-PG NPs showed 2.6-fold higher toxicity to breast cancer cells than fibroblast cells. The cell viability of breast cancer cells was reduced to ~80%, even at an initial concentration of 20 μg/mL. This might be due to the influence of a function in a NP component that might be specific to cancer cells. In cancer cell progression, it has been observed that cells show metabolic profile changes according to their adenosine triphosphate (ATP) requirements, such as an increased uptake rate of glucose for survival and cell multiplication under the cancer microenvironment [24,79]. The repeating unit of chitosan or modified chitosan consisting of a glucosamine unit, which mimics the glucose structure, may be one of the possibilities that is specific to cancer cells. This is suspected to be one of the reasons why DC-WCS-PG NPs underwent faster cellular uptake and inhibited the growth of cancer cells more than they did normal cells [80].

## 4. Conclusions

In this work, we demonstrated that using a water-soluble type of chitosan (WCS) with robust grafted poly(PEGMA) comb-like brushes (which was achieved using green water radiolysis-mediated graft copolymerization) significantly enhances water solubility and the dispersion of amphiphilic chitosan-based NP products. Optimizing water-based systems provides versatile synthetic processes for the preparation of amphiphilic nanoparticles in aqueous solution for drug-controlled release applications. The particle sizes of DC-WCS-PG NPs were 91 ± 9 nm. By using a water-soluble type of chitosan, high moieties in hydrophobic DC and hydrophilic poly(PEGMA) brushes can be achieved, with a high DS of ~98% and a DG of 252%, respectively. Due to the low MW of the WSC polymer backbone, the DS of DC molecules on WCS was found to be 3.5-fold higher than that on high-MW CS. This supports the drug encapsulation efficiency of DC-WCS-PG NPs, which was ~3.8-fold higher than that of DC-CS-PG NPs. It is also important to note that although high DC moieties were functionalized onto the NPs, the final DC-WCS-PG NPs product still showed good water dispersion at concentrations as high as 5 mg/mL. Both WCS and the robust grafted poly(PEGMA) brushes showed a synergistic component effect that improved the water dispersion of the final DC-WCS-PG NPs product. The DC-WCS-PG NPs efficiently encapsulated PTX and BBR water-insoluble drugs at loading efficiencies of 359.2 and 374.6 mg/g. The DC-WCS-PG NPs exhibited good characteristics that make them appealing for use as nanocarriers in drug delivery applications. The amphiphilic DC-WCS-PG NPs from the developed building blocks of WCS graft copolymer are a promising nanocarrier platform for (i) improved dispersion of NP products in water, (ii) enhanced water-insoluble drug encapsulation, (iii) desirable controlled release behavior, and (iv) reduced side effects from conventional drugs.

## Data Availability

Not applicable.
